# Genetic Ethnic Differences in Human 2′-5′-Oligoadenylate Synthetase and Disease Associations: A Systematic Review

**DOI:** 10.3390/genes14020527

**Published:** 2023-02-19

**Authors:** Anmol Gokul, Thilona Arumugam, Veron Ramsuran

**Affiliations:** 1 School of Laboratory Medicine and Medical Sciences, College of Health Science, University of KwaZulu-Natal, Durban 4041, South Africa; 2 Centre for the AIDS Programme of Research in South Africa (CAPRISA), University of KwaZulu-Natal, Durban 4001, South Africa

**Keywords:** human genetics, polymorphisms, infectious diseases, viral disease, OAS, SARS-CoV-2

## Abstract

Recently, several studies have highlighted a skewed prevalence of infectious diseases within the African continent. Furthermore, a growing number of studies have demonstrated unique genetic variants found within the African genome are one of the contributing factors to the disease severity of infectious diseases within Africa. Understanding the host genetic mechanisms that offer protection against infectious diseases provides an opportunity to develop unique therapeutic interventions. Over the past two decades, several studies have linked the 2′-5′-oligoadenylate synthetase (OAS) family with a range of infectious diseases. More recently, the *OAS-1* gene has also been associated with disease severity caused by the severe acute respiratory syndrome coronavirus 2 (SARS-CoV-2), which led to a global pandemic. The OAS family serves as an antiviral factor through the interaction with Ribonuclease-Latent (RNase-L). This review explores the genetic variants observed within the *OAS* genes and the associations with various viral infections and how previously reported ethnic-specific polymorphisms drive clinical significance. This review provides an overview of *OAS* genetic association studies with a particular focus on viral diseases affecting individuals of African descent.

## 1. Introduction

Genetic mutations and variations located within specific genes in the human genome have been found to associate with the outcome of various viral infections [1,2,3,4,5,6,7,8]. The human 2′-5′-oligoadenylate synthetase (OAS) family is one such set of genes shown to have genetic mutations associated with several viral infections [7,8,9,10,11,12,13,14,15,16]. The importance of oligoadenylate synthetase–Ribonuclease-Latent (OAS–RNase-L) in innate antiviral immunity has been highlighted by recent and exciting advances [17]. Antiviral proteins from the OAS family are type I and type II interferon (IFN)-induced antiviral proteins [18,19]. The OAS family’s antiviral activity is mediated by the activation of Ribonuclease-Latent (RNase-L) by sensing the presence of double-stranded Ribonuclease acid (RNA) [20]. The activation of RNase-L through the binding of 2′-5′ Adenylate (2-5A) and dimer formation has been identified and described within the past decade [21,22]. OAS–RNase-L initiates its antiviral activities via three antiviral processes, which can stop viruses from reproducing [23]. Firstly, OAS–RNase-L prevents viral reproduction by cleaving viral genomic single-stranded RNA (ssRNA) [23,24]. Cleavage of viral messenger-RNA (mRNA) to prevent viral protein synthesis is the second mechanism of suppression [23,24,25]. Finally, the cellular cleavage of mRNA and ribosomal ribonucleic acid (rRNA) is essential for viral replication [23]. In an attempt to counteract the OAS–RNase-L activity, some viruses have evolved a variety of anti-OAS–RNase-L strategies, such as sequestering double-stranded Ribonuclease acid (dsRNA) from OAS, degrading 2-5A with viral or host-encoded enzymes [23,26,27,28,29,30,31].

Studies have shown that the presence or absence of single nucleotide polymorphisms (SNPs) affect the outcome of severe clinical illness or long-term virological response [14,15,32]. Infectious disease susceptibility, severity, and overall clinical outcomes may all be influenced by host genetic diversity [33,34]. Individuals of various racial and ethnic backgrounds are known to have distinct allelic variations and frequencies [1,35]. It is these ethnic-specific variations, observed within causal disease-associated SNPs, that could drive the differences seen in the prevalence of infectious diseases across racial backgrounds [36,37]. SNPs located within the *OAS* genes have demonstrated an influence on viral infection susceptibility and disease severity [7,12,13,38,39,40,41].

Africa possesses the largest genomic diversity in the world; however, despite the rich genetic variability that exists within the continent, infectious disease associations are under-studied [35]. The rates of several viral infections, such as African swine fever virus, Dengue virus, Ebola virus, human immunodeficiency virus (HIV), Hepatitis B (HBV), Hepatitis C (HCV), West Nile virus (WNV), and Zika virus, are the highest in Africa compared to the rest of the world [42]. The majority of polymorphisms showing clinical significance are conducted within individuals of Caucasian ancestry [43]. SNPs are often used as markers to identify differences among individuals or populations [44,45]. These variations can have a range of effects on the regulation and function of the gene and can be used to study the genetic bias of traits and diseases [46,47]. In comparison to other populations, Africa exhibits higher levels of haplotype diversity, which may have an effect if multiple mutations are combined within a chromosomal region, lower levels of linkage disequilibrium (LD), more diverse LD patterns, and more complicated patterns of population substructure [35,48]. However, LD decays rapidly in African populations compared to non-African populations, thus resulting in far shorter haplotypes among the African population [35]. This review focuses on polymorphisms within the OAS family that vary across ethnic groups and show an association with viral diseases.

## 2. Polymorphisms within the *OAS* Family Associated with Viral Diseases

**Figure 1:**
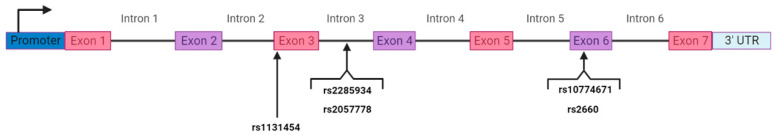
*OAS-1* gene with the location of SNPs from this study.

**Figure 2:**
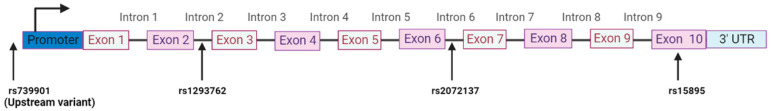
*OAS-2* gene with the location of SNPs from this study.

**Figure 3:**
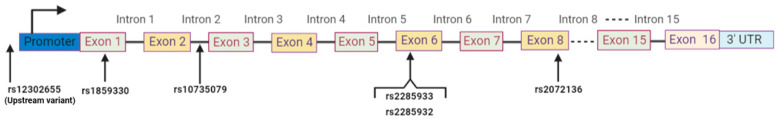
*OAS-3* gene with the location of SNPs from this study.

**Figure 4:**
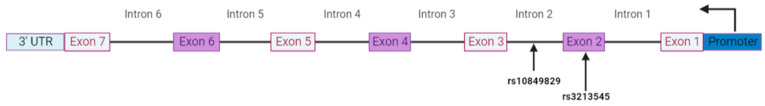
*OAS-L* gene with the location of SNPs from this study.

Using the search terms “*OAS-1, OAS-2, OAS-3* and *OAS-L*”, on the National Centre for Biotechnology Information, https://www.ncbi.nlm.nih.gov/ (accessed on 9 May 2022), we identified a list of publications that showed associations with polymorphisms located within the gene-regions of the abovementioned genes. A total of 77 SNPs were identified from publications (*OAS-1*; n = 29, *OAS-2*; n = 12, *OAS-3*; n = 23, *OAS-L*; n = 13). The inclusion criteria for SNPs selected consisted of studies reporting clinical significance for the variant within humans, publications that were in English, and polymorphisms with variable frequencies. The exclusion criteria were as follows: SNPs with a frequency of one or zero, data generated from computational analysis, SNP redundancy, and other specific reasons were noted. After implementation of the inclusion and exclusion criteria, a total of 17 SNPs were selected for discussion, i.e., *OAS-1*; n = 5, *OAS-2*; n = 4, *OAS-3*; n = 6, *OAS-L*; n = 2. Within the *OAS-1*, the following SNPs are discussed rs2057778 (G > T), rs2285934 (T > G), rs1131454 (G > A), rs10774671 (G > A), andrs2660 (G > A), (Table 1 and Figure 1); *OAS-2* SNPs that are discussed include rs1293762 (T > G), rs15895 (A > G), rs739901 (C > A), and rs2072137 (T > C) (Table 2 and Figure 2). SNPs within the OAS-3 gene that are reported on are rs1859330 (G > A), rs10735079 (G > A), rs2285932 (T > C), rs2072136 (G > A), rs2285933 (C > G), and rs12302655 (G > A) (Table 3 and Figure 3). Finally, we discuss rs3213545 (G > A) and rs10849829 (G > A) for *OAS-L* (Table 4 and Figure 4).

### 2.1. OAS-1

SNP rs2057778 (*OAS-1*; G > T) is an intron variant located within an interferon signalling pathway in intron 3. By interfering with the identification of the splice location, intronic variants could possibly affect alternative splicing, thus resulting in a defective protein [49]. rs2057778 (*OAS-1*; G > T), has been associated with chronic HCV progression, necroinflammatory activity and severe acute respiratory syndrome coronavirus 2 (SARS-CoV-2) [50,51]. As noted in Table 1, the frequency of this SNP varies across ethnicities, i.e., the MAF in Europeans (G-0.34), Africans (G-0.06), and Asians (G-0.19). rs2285934 (*OAS-1;* T > G) is an intronic variant that is involved in post-transcriptional gene control mediated by RNA binding proteins and is also located within intron 3. This SNP has been associated with HBV, liver disease in HIV/HCV-coinfected patients, WNV, and prostate cancer [50,52]. As noted in Table 1, the frequency of this SNP varies across ethnicities, i.e., the MAF in Europeans (T-0.33), Africans (T-0.50), and Asians (T-0.23). Within exon 3, SNP rs1131454 (*OAS-1*; G > A), formerly called rs3741981, is a synonymous SNP that results in an amino acid change from glycine to serine. While amino acid changes can alter a protein’s structure, they may not always alter protein function; however, mutations within conserved regions may possibly bring about a change in both structure and function of the protein [53]. rs1131454 (*OAS-1*; G > A) is involved in enterovirus, coronary atherosclerosis, tuberculosis (TB), HCV, Dengue virus, H1N1 and H5N1 influenza virus, WNV, and prostate cancer [7,38,54,55,56]. As noted in Table 1, the frequency of this SNP varies across ethnicities, i.e., the MAF in Europeans (G-0.43), Africans (G-0.77), and Asians (G-0.43). SNP rs2660 (*OAS-1*; G > A) is found in exon 6, and the SNP results in an Arg397Gly amino acid substitution, resulting in STOP gained and further resulting into a truncated protein (Zhao et al., 2013). rs2660 (*OAS-1*; G > A) participates in enterovirus71 (EV-71) infection, HCV, prostate cancer, and SARS-CoV-2 [12,54,57]. As noted in Table 1, the frequency of this rs2660 (*OAS-1*; G > A) varies across ethnicities, i.e., the MAF in Europeans (G-0.36), Africans (G-0.09), and Asians (G-0.23). Figure 1 represents the location of the SNPs within the *OAS-1* gene.

Several *OAS-1* SNPs (rs2057778, rs2285934, rs1131454, rs10774671, and rs2660) were associated with HBV or HCV infection (Table 1, Figure 1). In a population of European descent, López-Rodríguez et al. (2017) reported rs2057778 (*OAS-1*; G > C) to be significantly associated with severe necro-inflammatory activity in chronic HCV [50]. rs2057778 (*OAS-1*; G > T), showed increased risk to SARS-CoV-2 [51].

The rs2285934 (*OAS-1;* T > A, Figure 1) polymorphism is located within an enhancer region, and it is thought that the presence of the “A” allele alters the expression levels of *OAS-1* [52]. The higher frequency of the “A” allele in rs2285934 (*OAS-1;* T > A) was shown to associate with severe liver disease in an HIV- and HCV-coinfected population of European descent in a cross-sectional study of 219 patients [52]. Furthermore, rs2285934 (*OAS-1;* T > A) appears to be involved in RNA binding protein-mediated post-transcriptional gene regulation based on in silico experiments, which may increase *OAS-1* expression, leading to better HCV control in HIV-infected individuals [52]. The genetic variation observed within the *OAS-1* gene region is considered to have a significant impact on the effectiveness of the OAS–RNase-L pathway, which might be a predictor for the advancement of liver disease in HCV infection [50].

Asian individuals with chronic HCV were shown to have significantly higher frequencies of the “AA” and “AG” genotypes within the rs3741981 variant, which has been merged into SNP rs1131454 (*OAS-1*; G > A, Figure 1), and this locus is now considered to be a genetic risk factor for HCV disease shown in a Chinese study consisting of 603 patients (Controls n = 305; HCV positive n = 298) [54]. A Vietnamese study investigating severe acute respiratory syndrome coronavirus 1 (SARS-CoV-1) revealed that higher frequencies of the “G” allele of rs1131454 (*OAS-1*; G > A) affected susceptibility and progression [56]. The frequency “GA” and “GG” genotypes of rs1131454 (*OAS-1*; G > A) were also found to be higher in 44 Vietnamese severe acute respiratory syndrome (SARS) patients (Controls n = 153; SARS positive n = 44) [7]. This was confirmed by Tanimine et al. (2021) in a study that consisted of 230 patients that showed that the rs1131454 (*OAS-1*; G > A) was associated with disease severity in SARS-CoV-2 patients in a Vietnamese population [55]. The increased frequency of the “A” allele in rs1131454 (*OAS-1*; G > A) was shown to be associated with severe SARS-CoV-2 disease in Europeans; however, in a smaller study, the polymorphism showed no significant association [38]. In contrast, Banday et al. (2021) suggested that the presence of rs1131454 (*OAS-1*; G > A) and rs10774671 (*OAS-1*; G > A) contributed to the impaired clearance of SARS-CoV-2 in Europeans when analyzing 266 genotyped patients [38].

The rs10774671 (*OAS-1*; G > A, Figure 1) polymorphism plays an important role in the functioning of the *OAS-1* gene, as it serves as a splice-acceptor site for exon 7 [54]. The presence of the “G” allele results in splicing, which produces a p46 form with high enzymatic OAS activity. The presence of the “A” allele at this location results in two mRNA variants, p48 and p52, both of which are linked with decreased OAS enzymatic activity [54,58]. rs10774671 (*OAS-1*; G > A) has been involved in TB, HBV, HCV, tick-borne encephalitis (TBE), WNV, and severity of hand, foot, and mouth disease, and more recently, SARS-CoV-2 [38,54,58,59,60,61]. As noted in Table 1, the frequency of this SNP varies across ethnicities, i.e., the MAF in Europeans (G-0.36), Africans (G-0.57), and Asians (G-0.25).

The “AG” and “GG” genotypes of rs10774671 (*OAS-1*; G > A) was significantly higher among 298 HCV Asian patients in comparison to a control group (n = 305) [54]. Increased resistance to HCV has been linked to the Neandertal-like haplotype [62]. The Neandertal-like splice acceptor variation of the “G” allele found in rs10774671 (*OAS-1*; G > A) has been linked to protection against WNV [58]. In another study conducted among 1053 Caucasians (Controls n = 552; WNV positive n = 501) from five United States Centers, an increase in the “A” allele of rs10774671 (*OAS-1*; G > A) was identified as a host genetic risk factor for initial infection with WNV [58]. An investigation into Taiwanese children (Controls n = 163; positive patients n = 333) that suffered from hand, foot and mouth disease, human enterovirus, and coxsackie virus revealed that children with the homozygous “GG” genotype of rs10774671 (*OAS-1*; G > A) were susceptible to coxsackie virus infection, while children that suffered with hand, foot and mouth disease, and had a “AA” and “AG” genotype, were more prone to develop encephalitis [61]. In another study involving 198 Chinese patients (Controls n = 71; HBV positive n = 126), the relationship between rs10774671 (*OAS-1*, G > A) and hepatitis B e-antigen (HbeAg) revealed that an increase in the frequency of the “G” allele benefited patients with chronic HBV infection to achieve HbeAg seroconversion [60]. Regarding rs10774671 (*OAS-1*; G > A), a genome-wide association (GWAS) study discovered a protective haplotype variant against severe SARS-CoV-2 that was found to be similar to that present in all three Neanderthal genomes [63]. However, variation in the haplotype was connected to becoming severely ill with COVID-19 when infected with SARS-CoV-2 [63]. To better understand the role of the antiviral defenses of *OAS*, Wickenhagen et al. (2021) showed that the “G” allele, rs10774671 (*OAS-1*; G > A), resulted in a prenylated *OAS-1* form that was associated with protection from severe SARS-CoV-2 infection in a lung cell line [64]. rs10774671 (*OAS-1*; G > A) ”A” allele is regarded as a risk allele in Europeans (Controls n > 1,000,000; positive n = 24,876), and it was suggested that the *OAS-1* deficiency could be an underlying risk for severe SARS-CoV-2 disease, while the “G” allele produces a protective p46 isoform of *OAS-1* in Africans (Controls n = 130,997; Combined positive patients n = 2787) [59,65].

rs10774671 (*OAS-1*; G > A) and rs2660 (*OAS-1*; G > A) are in perfect LD in Europeans and Asians; this is not true within the African population [38]. It was observed the frequency distributions of rs10774671 (*OAS-1*; G > A) and rs2660 (*OAS-1*; G > A) within the Chinese population differed from European, Japanese, and Vietnamese populations [54]. SNP rs2660 (*OAS-1*; G > A, Figure 1) is found in exon 6, and the SNP results in an Arg397Gly amino acid substitution, resulting in STOP gained and further resulting into a truncated protein (Zhao et al. 2013). An increase in “AG,” and “GG” genotype frequencies of rs2660 (*OAS-1*; G > A) were demonstrated to be genetic risk factors for chronic HCV infection. The heterozygous “AG” genotype was more likely to induce central nervous system damage after EV-71 infection, according to a study of Chinese children (Controls n = 201; EV71 positive n = 180) bearing SNP rs2660 (*OAS-1*; G > A) [12]. The Neandertal missense mutation in rs2660 (*OAS-1*; G > A) has been linked to moderate to substantial protection against SARS-CoV-2 infection [66]. A Chinese study indicated that an increase in the frequency of the “G” allele of rs2660 (*OAS-1*; G > A) was associated with a protective effect against SARS infection [56]. Individuals that have increased frequencies of the “A” allele may be likely to have poor SARS-CoV-2 disease outcomes in comparison to those who carry the “G” allele [67]. In a separate study, the Neanderthal haplotype, rs2660 (*OAS-1*; G > A), was shown to be associated with variable protection against SARS-CoV-2 infection, and in contrast, the polymorphism is associated with susceptibility to EV-71 infection in Chinese children (Controls n = 201; Combined positives n = 2914) [12,59,66].

### 2.2. OAS-2

rs1293762 (*OAS-2*; T > G) is an intron variant located in intron 2, which may play a role in RNA alternative splicing or affect transcription [68]. rs1293762 (*OAS-2*; T > G) is involved in tick-borne encephalitis (TBE), Dengue virus, and HCV [69]. As noted in Table 2, the frequency of this rs1293762 (*OAS-2*; T > G) varies across ethnicities, i.e., the MAF in Europeans (T-0.43), Africans (T-0.15), and Asians (T-0.08). The “A” allele in rs15895 (*OAS-2*; A > G), located in exon 9, changes the tryptophan codon 720, which is a deletion of 8 amino acids resulting in a translational stop codon and thus a truncated protein [69]. This SNP has been associated with the Dengue virus and TBE [58,69,70,71]. As noted in Table 2, the frequency of this rs15895 varies across ethnicities, i.e., the MAF in Europeans (A-0.35), Africans (A-0.08), and Asians (A-0.004). rs739901 (*OAS-2*; C > A) is an upstream variant and may play a regulatory role of the *OAS-2* gene and has been associated with enterovirus [57]. The MAFs of the SNP differ across ethnicities, i.e., in Europeans (C-0.81), Africans (C-0.88), and Asians (C-0.61). Found in intron 6 is rs2072137 (*OAS-2*; T > C), which may play a role in RNA alternative splicing or affect transcription [68] and has been linked to HIV and variable activity of the *OAS-2* gene [14]. As noted in Table 2, the frequency of this rs2072137 varies across ethnicities, i.e., the MAF in Europeans (T-0.59), Africans (T-0.88), and Asians (T-0.57). Figure 2 showcases the SNP positions within the *OAS-2* gene.

Two SNPs found in *OAS-2* (rs1293762 and rs15895) have been associated with TBE and Dengue fever (Table 2, Figure 2). In a study within Eurasia made up of Caucasians (Study group n = 873) from Russia and Germany, Central Asian Mongolians, and Arctic Mongolians, rs1293762 (*OAS-2*; T > G), (Figure 2) was linked to disease outcome in patients with TBE, while an increase in the frequency of the “A” allele of rs15895 (*OAS-2*; A > G) was found to be significantly higher in severe TBE patients [71]. It was concluded that there were differences among the genotype, allele, and haplotype frequencies [71]. The SNP also demonstrated an increase in frequency associated with differential susceptibility to Dengue virus and a predisposition to chronic HCV [52,69,72,73]. rs1293762 (*OAS-2*; T > G, Figure 2), appears also to be involved in RNA binding protein-mediated post-transcriptional gene regulation in the in silico investigation and increases the expression of *OAS-2* leading to better HCV control in HIV infected individuals [52].

In rs15895 (*OAS-2*; A > G, Figure 2), increased “GG” genotype frequencies were shown to be associated with differential susceptibility to Dengue virus (Controls n = 105; DV positive n = 109) and were also associated with TBE (Controls n = 302; TBE positive n = 142), in an Indian and Russian study, respectively [69,70].

rs739901 (*OAS-2*; C > A, Figure 2) is involved in the susceptibility to EV-71 infection, and it was suggested that an increase in the frequencies of the “A” allele may play a role in the regulation of IFN-γ production in EV-71 infection within Chinese children (Controls n = 252; EV-71 positive n = 294) [57].

SNP rs2072137 (*OAS-2*; T > C, Figure 2) has been reported to be associated with HIV disease progression and an increase of frequencies of the homozygous “TT” genotype related to the slow progression of HIV disease in a Russian study that comprised of 94 HIV positive patients [14].

### 2.3. OAS-3

rs1859330 (*OAS-3*; G > A) is a missense variant located within the coding sequence of exon 1 and is associated with E-71 infection and HIV progression [14,74]. SNPs that are located within the coding region of the *OAS* gene may or may not change the amino acid sequence of the protein [75]. As noted in Table 3, the frequency of this rs1859330 (*OAS-3*; G > A) varies across ethnicities, i.e., the MAF in Europeans (G-0.37), Africans (G-0.43), and Asians (G-0.25). rs10735079 (*OAS-3*; G > A) is an intron variant located within intron 2 and has been associated with SARS-CoV-2 and lymphoma [76,77]. As noted in Table 3, the frequency of rs10735079 (*OAS-3*; G > A) varies across ethnicities, i.e., the MAF in Europeans (G-0.37), Africans (G-0.23), and Asians (G-0.20). rs2285932 (*OAS-3*; T > C), located within the coding sequence of exon 6, results in an amino acid substitution (Ile438Ile) that may influence pre-messenger RNA splicing regulation [69]. There are reports of a haplotype effect that is associated with the Dengue virus [70]. Table 3 notes the MAF variability across ethnicities, i.e., the MAF in Europeans (T-0.33), Africans (T-0.07), and Asians (T-0.09). rs2072136 (*OAS-3*; G > A) is located within the coding sequence of exon 8, resulting in an amino acid substitution (Ser567Ser) that may influence pre-messenger RNA splicing regulation [69]. rs2072136 (*OAS-3*; G > A) has been associated with TBE, HBV, and a haplotype effect within the Dengue virus [69,70,71,73,78]. As noted in Table 3, the frequency of this rs2072136 (*OAS-3*; G > A) varies across ethnicities, i.e., the MAF in Europeans (G-0.77), Africans (G-0.88), and Asian (G-0.37). rs2285933 (*OAS-3*; C > G) is located within the coding sequence of exon 6 and has been associated with the Dengue virus [79]. Table 3 notes the differences in the MAF, with the disparity displayed among the Asian population, i.e., the MAF in Europeans (C-0.75), Africans (C-0.75), and Asians (C-0.83). rs12302655 (*OAS-3*; G > A) is an upstream variant that has been associated with HPV [80]. Although Table 3 notes the MAF for the Europeans and Asians being remarkably similar, there is a disparity within the African population, i.e., the MAF in Europeans (G-1.0), Africans (G-0.71), and Asians (G-0.99). The location of the abovementioned SNPs is located in Figure 3.

rs1859330 (*OAS-3*; G > A, Figure 3) is a missense variant, and a frequency increase of the “A” allele polymorphism is a risk factor for severe development of EV-71 in Chinese Han children (Controls n = 344; VE-71 positive n = 370) [74]. There is mounting evidence that the innate immune system plays a key role in HIV infection and progression and that immune pathway polymorphisms may influence susceptibility and progression [81]. The *OAS-3* variant rs1859330 (G > A) has a strong link to HIV disease progression in a Russian study involving Europeans (HIV positive n = 94). It was discovered that the homozygous “AA” genotype was linked to delayed illness progression, whereas the “GG” and “GA” genotypes were linked to “normal” disease progression [14].

In rs10735079 (*OAS-3*; G > A, Figure 3), increased frequency of the “A” allele is linked to the possibility of the promotion of SARS-CoV-2 Alzheimer’s patients from the United Kingdom (Controls n = 1234; Alzheimer patients n = 7417) [76,77].

Two SNPs from *OAS-3* (rs2285932 and rs2072136) were associated with HBV disease (Table 3, Figure 3). The abovementioned SNPs, in addition to others (rs2285932, rs2072136, and rs2285933), are also associated with the Dengue virus. Whole exome sequencing found sixteen rare variants in the protein-coding region of *OAS-3* in individuals chronically infected with HBV. These *OAS-3* polymorphisms were associated with the failed clearance of hepatitis B-antigen (HBAg) in the presence of anti-HB. The variation in the *OAS-3* gene may have affected expression or activity, leading to inefficient activation of *RNase-L* [82]. An increase in “TA” genotype frequency of the missense SNP rs2285932 (*OAS-3*; T > A), and an increase in the “GA” genotype frequency of synonymous variant rs2072136 (*OAS-3*; G > A) are associated with the susceptibility to clinical outcomes in HBV within an Indian population from Pune [69,70,78].

An increase in the frequency of the “T” allele of the missense SNP rs2285932 (*OAS-3*; T > A, Figure 3) was associated with lower susceptibility to clinical outcomes in Dengue virus infection and synonymous variant rs2072136 (*OAS-3*; G > A), “A” allele was associated with the lower outcome of TBE virus and haplotypes are associated with an Indian population from Pune (Controls n = 978; Combined Dengue virus and TBE n = 161) [69,70,78]. This was confirmed in another study that confirmed increases in frequencies of the “TT” genotype in rs2285932 (*OAS-3*; T > A), and the “GG” genotype of rs2072136 (*OAS-3*; G > A) was found in patients with severe TBE [71]. Despite the fact that both rs2285932 (*OAS-3*; T > A) and rs2072136 (*OAS-3*; G > A) do not cause amino acid alterations, they may have an impact on pre-mRNA splicing regulation in the TBE virus [69]. According to findings gathered from the research of *OAS* gene SNPs, variation in this component of the innate immune response may have a role in the outcome of a TBE virus infection [69]. rs2285932 (*OAS-3*; T > C) and rs2072136 (*OAS-3*; G > A) C-G haplotype has shown some significance in the association to reduced risk of Dengue virus within an Asian population (Controls n = 105; Dengue virus positive n = 109) [70].

In rs2285933 (*OAS-3*; C > G, Figure 3), the increased frequency of the “G” allele was shown to have a protective effect in Dengue haemorrhagic fever amongst the sub-Saharan African populations, while the opposite was seen in Northeast and Southeast Asians [79].

Persistent HPV infection can sometimes lead to the development of cervical intraepithelial neoplasia and, eventually, cervical cancer. A study looking at HPV progression to cervical cancer (Controls n = 492; HPV persistence and cancer n = 616) found that rs12302655 (*OAS-3*, G > A) had a significant association with HPV persistence and disease progression [80].

### 2.4. OAS-L

rs3213545 (*OAS-L*; G > A) found within the coding sequence of exon 2 and has been associated with WNV and HCV [15,83,84,85]. Table 4 highlights the MAFs of rs3213545 (*OAS-L*; G > A), i.e., the MAF in Europeans (G-0.71), Africans (G-0.87), and Asians (G-0.53). rs10849829 (*OAS-L*; G > A) found within intron 2 of the *OAS-L* gene and has been linked to an interferon response in the presence of HCV infection [78]. The MAFs of rs10849829 (*OAS-L*; G > A) across ethnicities differ, i.e., the MAF in Europeans (G-0.58), Africans (G-0.80), and Asians (G-0.24), as per Table 4. Figure 4 1 represents the location of the SNPs within the *OAS-L* gene.

Both SNPs found in Table 4, Figure 4 (rs3213545 and rs10849829) have been associated with Hepatitis infection (Combined Controls n = 552; Hepatitis positive n = 668) [15,78,83,84]. Polymorphisms in the *OAS-L* gene, particularly the missense variant rs3213545 (G > A, Figure 4), have been shown to play a role in the susceptibility to WNV (Combined Controls n = 129; combined WNV positives n = 440) and are also associated with chronic HCV (HCV positives n = 52) [83,84,85]. In a group of patients (consisting of 27 Caucasian, 4 African American, and 2 Hispanic) infected with WNV, the OAS family (exons) was sequenced; the presence of rs3213545 (*OAS-L*; G > A) was associated with WNV (Controls n = 60; WNV positive n = 33) infection susceptibility in the Caucasian population, noting that only the Caucasian data were utilized for analysis [84]. The low sample numbers in the study may not be enough to justify their results. The link to WNV is due to the presence of a minor “T” allele in the splicing enhancer region, which results in a dominant-negative mutant version of *OAS-L* [15]. In contrast, the minor “T” allele in rs3213545 (*OAS-L*; G > A) has been significantly associated with a sustained virological response in HCV infection (Combined HCV positive n = 401) [15,83].

Caucasian children affected with chronic HBV (HBV positive n = 52) were treated with pegylated interferon, and it was reported that the homozygous “AA” genotype for rs10849829 (*OAS-L*; G > A, Figure 4) was frequently found in patients that did not respond to IFN treatment [78].

## 3. The African Paradox

Changes in the *OAS* genes can influence the outcome of an illness, and SNPs present within the *OAS* family have been linked to the susceptibility and severity of infectious illnesses and autoimmune diseases [9,16,38].

This review outlines the need for additional research into what African diversity and genome have to offer. When investigating the average nucleotide diversity among Europeans, Asians, and Africans, it was noted that the diversity was almost twice as high among Africans [86]. From what we have observed in Table 1, Table 2, Table 3 and Table 4, there has not been sufficient research conducted within the African setting. Most published data have shown the significant role OAS plays in disease within European and Asian populations [35]. Furthermore, drastic differences have been reported in the frequency of certain OAS SNPs within African populations compared to European and Asian populations, and this can be observed within Table 1, Table 2, Table 3 and Table 4 with variable SNP frequencies between European and African ethnicities across the entire OAS family. rs2057778 (*OAS-1*; G > T), rs1131454 (*OAS-1*; G > A), rs2660 (*OAS-1*; G > A), rs15895 (*OAS-2*; A > G), rs2285932 (*OAS-3*; T > C), rs12302655 (*OAS-3*; G > A) highlights a MAF difference greater than 30% between Europeans and Africans, respectively. Interestingly, from our observation, none of the abovementioned SNPs have data associated with any viral diseases. Another aspect that could be driving disease severity and susceptibility could be associated with SNPs not being in LD across ethnicities. Africa has one of the highest prevalence rates of infectious diseases, but there is a lack of data evaluating the role of OAS-associated SNPs in the African population [42,87,88]. Evaluating the prevalence and understanding the role of these OAS-associated variants within an African context may help curb the disparity of infectious diseases within Africa.

## 4. Conclusions

Understanding the impact of SNPs and the levels of expression change as the disease progresses within various ethnic groups is critical. Our research agenda is influenced by significant knowledge gaps in viral and host genetics. Conducting candidate gene, epigenetic, and GWAS research across various racial and ethnic communities to find genes and haplotypes linked to various infection-related variables and clinical outcomes. Studies of any differences in the severity of disease caused by viral mutations and recombination events in relation to host comorbidities and the use of drugs that block, reduce, or promote differences in host gene expression need to be conducted.

There are significant gaps in knowledge among individuals of African descent, and SNPs that are associated with individuals of European or Asian descent may not associate with those from Africa. This is problematic since Africa has the highest rate of infectious diseases in the world [42,87,88]. Future studies need to be directed toward understanding the rich diversity within the African continent. Furthermore, studies need to focus on African-specific mutations and the role that these mutations play a role within an African setting. This review highlights the importance of this type of research. *OAS* is an extremely important gene, as demonstrated by its role in infectious diseases in other ethnicities. However, the role of the *OAS* gene and SNPs within the gene are under-studied within the African setting.

## Figures and Tables

**Table 1:** List of polymorphisms within the *OAS-1* gene associating with infectious diseases across ethnicities.

*OAS-1*	European	African	Asian	References	Mechanism
rs2057778 (G > T)	Hepatitis C			[50]	Located within an interferon signalling pathway
	SARS-CoV-2			[51]	
	G-0.34; 15 670	G-0.06; 3 622	G-0.19; 160	* NCBI (MAF; n=)	
rs2285934 (T > G)	Severe liver disease in HIV			[52]	Involved in post-transcriptional gene control mediated by RNA-binding proteins
	Hepatitis C			[52]	
	T-0.33; 15 646	T-0.50; 3 618	T-0.23; 160	* NCBI (MAF; n=)	
rs1131454 (G > A)			Hepatitis C	[54]	Missense variant
	SARS-CoV-2		SARS-CoV-2	[7,38,55,56]	
	G-0.43; 209 326	G-0.77; 5 334	G-0.43; 6 630	* NCBI (MAF; n=)	
rs10774671 (G > A)	West-Nile Virus			[58]	Splice acceptor
	SARS-CoV-2			[38]	
		SARS-CoV-2		[59]	
			Hepatitis B	[54,60]	
			Hand, Foot, and Mouth disease; Human Enterovirus, and Coxsackie virus	[61]	
			Hepatitis C	[54]	
	G-0.36; 256 676	G-0.57; 8 296	G-0.25; 6 854	* NCBI (MAF; n=)	
rs2660 (G > A)			Hepatitis C	[54]	Pre-mature stop codon
			Enterovirus	[12,57]	
	G-0.36; 260 764	G-0.09; 8 882	G-0.23; 6 898	* NCBI (MAF; n=)	

Minor Allele Frequencies (MAF) were recorded for each ethnic group using data obtained from the National Center for Biotechnology Information (NCBI), https://www.ncbi.nlm.nih.gov/snp, (accessed on 9 May 2022).

**Table 2:** List of polymorphisms within the *OAS-2* gene associating with infectious diseases across ethnicities.

*OAS-2*	European	African	Asian	References	Mechanism
rs1293762 (T > G)	Tick-borne encephalitis		Tick-borne encephalitis	[71]	Located near the centre of intron 2, in a splice enhancer or silencer element that has yet to be found
	Dengue Virus			[52,72,73]	
	Hepatitis C		Hepatitis C	[52,73]	
	T-0.43; 14 286	T-0.15; 2 946	T-0.08; 112	* NCBI (MAF; n=)	
rs15895 (A > G)	Dengue Virus		Dengue Virus	[70,71]	Translation stop codon when SNP is present
			Tick-Borne Encephalitis Virus	[58,69]	
	A-0.35; 303 624	A0.08; 8 566	A-0.004; 4 970	* NCBI (MAF; n=)	
rs739901 (C > A)			Enterovirus	[57]	Affects IFN-γ levels in EV71-infected patients
	C 0.81; 76 210	C-0.88; 7 574	C-0.61; 318	* NCBI (MAF; n=)	
rs2072137 (T > C)	HIV			[14]	Reduces the antiviral effect of IFN-α
	T-0.59; 182 916	T-0.88; 11 008	T-0.57; 802	* NCBI (MAF; n=)	

Minor Allele Frequencies (MAF) were recorded for each ethnic group using data obtained from the National Center for Biotechnology Information (NCBI), https://www.ncbi.nlm.nih.gov/snp/, (accessed on 9 May 2022).

**Table 3:** List of polymorphisms within the *OAS-3* gene associating with infectious diseases across ethnicities.

*OAS-3*	European	African	Asian	References	Mechanism
rs1859330 (G > A)			Enterovirus	[74]	Missense variant
	HIV progression			[14]	
	G-0.37; 73 116	G-0.43; 5 944	G-0.25; 6 308	NCBI (MAF; n=)	
rs10735079 (G > A)	SARS-CoV-2			[76,77]	Encode enzymes that produce a host antiviral mediator
	G-0.37; 207 624	G-0.23; 8 640	G-0.20; 3 846	NCBI (MAF; n=)	
rs2285932 (T > C)			Tick-borne encephalitis	[69,70,78]	May influence pre-messenger RNA (mRNA) splicing regulation
			Hepatitis B	[69,70,78]	
			Dengue Virus	[70]	
	T-0.33; 152 378	T-0.07; 7 984	T-0.09; 734	NCBI (MAF; n=)	
rs2072136 (G > A)			Tick-borne encephalitis	[69,70,78]	May influence pre-messenger RNA (mRNA) splicing regulation
			Hepatitis B	[70,73,78]	
			Dengue Virus	[70]	
	G-0.77; 151 456	G-0.88; 7 982	G-0.37; 734	NCBI (MAF; n=)	
rs2285933 (C > G)		Dengue Virus	Dengue virus	[79]	Contributes to pathogenesis in the process of blocking viral infection
	C-0.75; 94 924	C-0.75; 2 898	C-0.83; 3 302	NCBI (MAF; n=)	
rs12302655 (G > A)	Human papillomavirus			[80]	2KB upstream variant located within a regulatory
	G-1.0; 15 640	G-0.71; 4 520	G-0.99; 160	NCBI (MAF; n=)	

Minor Allele Frequencies (MAF) were recorded for each ethnic group using data obtained from the National Center for Biotechnology Information (NCBI), https://www.ncbi.nlm.nih.gov/snp/, (accessed on 9 May 2022).

**Table 4:** List of polymorphisms within the *OAS-L* gene associating with infectious diseases across ethnicities.

*OAS-L*	European	African	Asian	References	Mechanism
rs3213545 (G > A)	West-Nile virus			[15,83,85]	Splice enhancer site
	Hepatitis C			[15,83,84]	
	**G-0.71; 148,824**	**G-0.87; 7140**	**G-0.53; 646**	NCBI (MAF; n=)	
rs10849829 (G > A)	Hepatitis B			[78]	The AA genotype was associated with failure of IFN therapy
	**G-0.58; 69,492**	**G-0.80; 3752**	**G-0.24; 136**	NCBI (MAF; n=)	

Minor Allele Frequencies (MAF) were recorded for each ethnic group using data obtained from the National Center for Biotechnology Information (NCBI), https://www.ncbi.nlm.nih.gov/snp, (accessed on 9 May 2022).

## Data Availability

The data presented in this study were openly obtained from the National Center for Biotechnology Information (NCBI), https://www.ncbi.nlm.nih.gov/snp/, (accessed on 9 May 2022).
